# Supplementary material for: Recurrent Translocations in Topoisomerase Inhibitor-Related Leukemia Are Determined by the Features of DNA Breaks Rather Than by the Proximity of the Translocating Genes
Source: Int J Mol Sci. 2022 Aug 29;23(17):9824. doi: 10.3390/ijms23179824 (PMC9456246; doi:10.3390/ijms23179824)
Supplement: Supplementary file 1 [file ijms-23-09824-s001.zip › Figure S2. Correlation plot for replicates of 4C-seq experiments.pdf]

		non-treated		etoposide-treated		non-treated	
		AML1_1	*AML1_2*	*AML1_1*	*AML1_2*	*CCND1_1*	*CCND1_2*
AML1_1	non-treated	1	0,9999	0,9999	0,9999	0,0001	-0,0006
AML1_2	non-treated	0,9999	1	1,0000	1,0000	0,0001	-0,0006
AML1_1	etoposide-treated	0,9999	1,0000	1	1,0000	0,0001	-0,0005
AML1_2	etoposide-treated	0,9999	1,0000	1,0000	1	0,0001	-0,0006
CCND1_1	non-treated	0,0001	0,0001	0,0001	0,0001	1	0,9987
CCND1_2	non-treated	-0,0006	-0,0006	-0,0005	-0,0006	0,9987	1
CCND1_1	etoposide-treated	-0,0003	-0,0003	-0,0002	-0,0003	0,9986	0,9984
CCND1_2	etoposide-treated	-0,0005	-0,0005	-0,0005	-0,0005	0,9982	0,9968
MLL_1	non-treated	-0,0001	-0,0001	0,0000	-0,0001	0,0030	0,0074
MLL_2	non-treated	0,0005	0,0005	0,0006	0,0005	0,0034	0,0077
MLL_1	etoposide-treated	-0,0001	-0,0001	-0,0001	-0,0001	0,0031	0,0075
MLL_2	etoposide-treated	0,0004	0,0004	0,0004	0,0004	0,0038	0,0080

etoposide-treated		non-treated		etoposide-treated	
CCND1_1	*CCND1_2*	*MLL_1*	*MLL_2*	*MLL_1*	*MLL_2*
-0,0003	-0,0005	-0,0001	0,0005	-0,0001	0,0004
-0,0003	-0,0005	-0,0001	0,0005	-0,0001	0,0004
-0,0002	-0,0005	0,0000	0,0006	-0,0001	0,0004
-0,0003	-0,0005	-0,0001	0,0005	-0,0001	0,0004
0,9986	0,9982	0,0030	0,0034	0,0031	0,0038
0,9984	0,9968	0,0074	0,0077	0,0075	0,0080
1	0,9973	0,0038	0,0042	0,0040	0,0045
0,9973	1	0,0034	0,0038	0,0036	0,0042
0,0038	0,0034	1	0,9996	0,9993	0,9993
0,0042	0,0038	0,9996	1	0,9996	0,9991
0,0040	0,0036	0,9993	0,9996	1	0,9987
0,0045	0,0042	0,9993	0,9991	0,9987	1
