# Supplementary figures and images for: Recurrent Translocations in Topoisomerase Inhibitor-Related Leukemia Are Determined by the Features of DNA Breaks Rather Than by the Proximity of the Translocating Genes
Source: Int J Mol Sci. 2022 Aug 29;23(17):9824. doi: 10.3390/ijms23179824 (PMC9456246; doi:10.3390/ijms23179824)

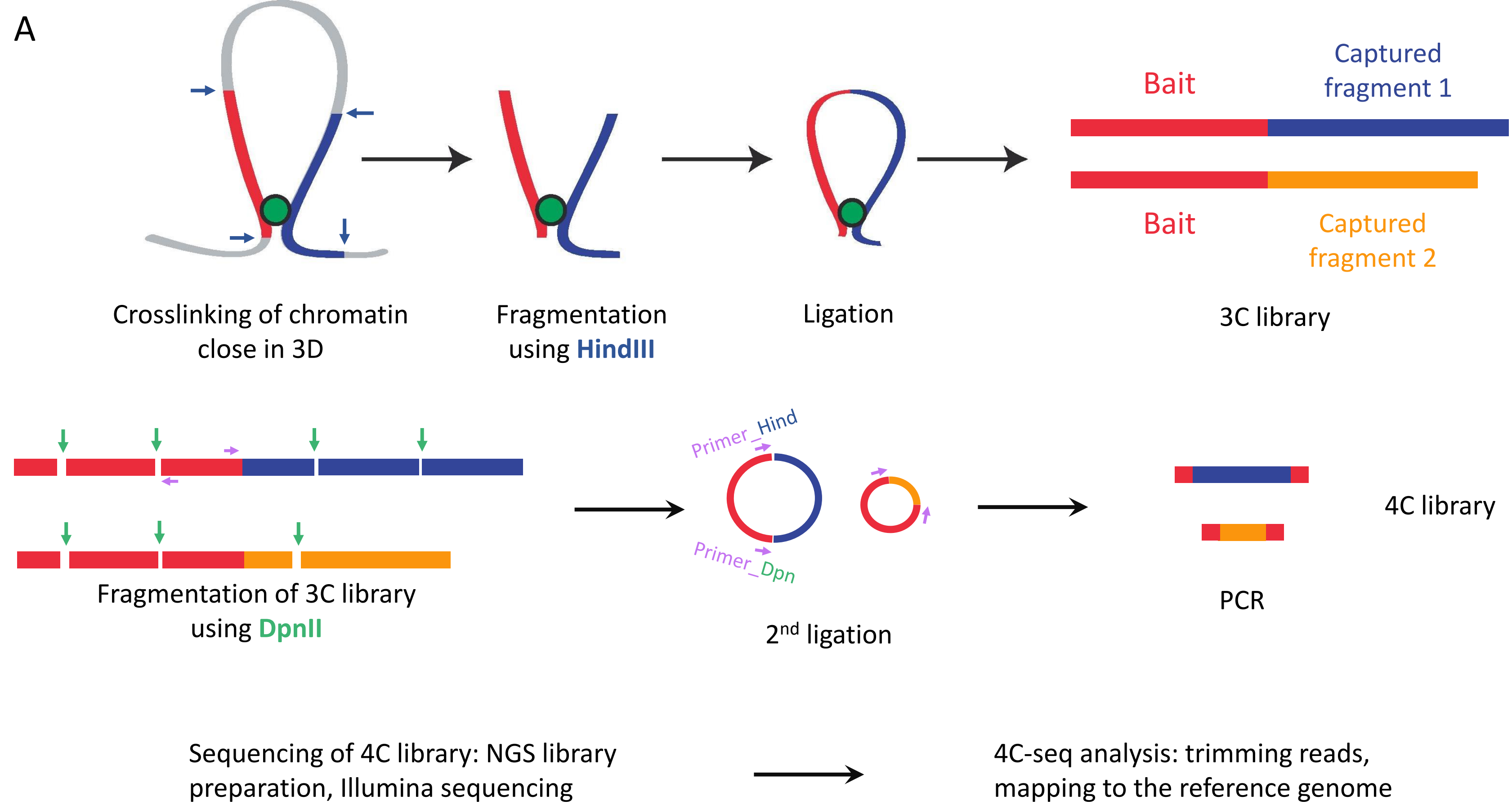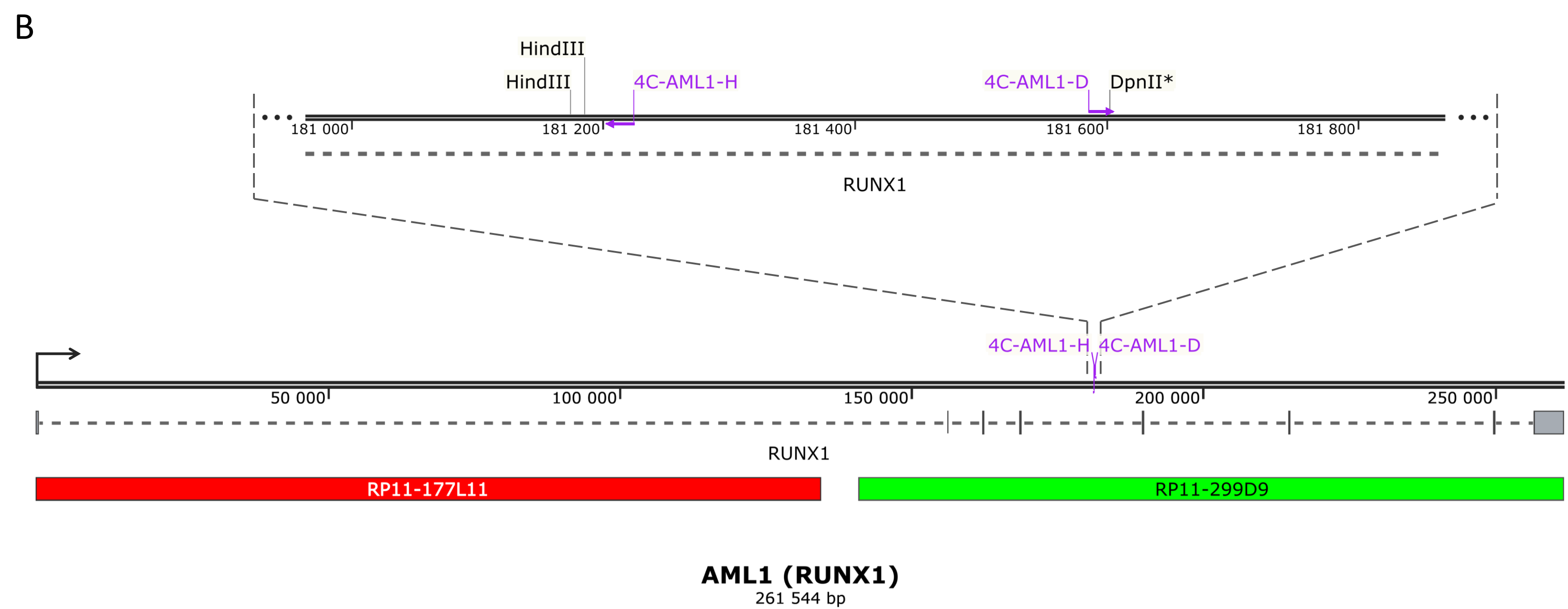

Supplement: Supplementary file 1 [file ijms-23-09824-s001.zip › Figure S1. 4C-seq workflow and localization of the 4C-seq primers and FISH probes.pdf]
